# Editorial: Epigenomics and metabolomics: pioneering new frontiers in breast cancer research and treatment

**DOI:** 10.3389/fonc.2026.1855583

**Published:** 2026-05-19

**Authors:** Purvi Purohit, Parmod Kumar, Anupama Modi

**Affiliations:** 1 Department of Biochemistry, All India Institute of Medical Sciences Jodhpur, Jodhpur, India; 2 Department of Medical Oncology Hematology, All India Institute of Medical Sciences Jodhpur, Jodhpur, India; 3 GTU-School of Applied Sciences and Technology, Ahmedabad, India

**Keywords:** breast cancer, epigenomics, metabolomics, precision medicine, TME (tumor microenvironment)

Breast cancer (BC) is a heterogeneous disease that has a unique pathology and genetic makeup for each subtype. As the tumor evolves, additional mutations contribute to its heterogeneity. Genomics plays a significant role in precision medicine for the targeted therapy of BC, with multiple drugs targeting distinct genetic mutations, ranging from the hormone receptor-targeting drug tamoxifen to the BRCA-targeting PARP inhibitors. However, epigenomics and metabolomics are emerging fields in which much remains to be done to develop targeted therapeutics. This Research Topic is a compilation of original studies on the theme of *“Epigenomics and Metabolomics in Breast Cancer: the new pioneering field”*, aimed at showcasing the best research in this field.

The mTOR pathway is a crucial regulator of epithelial-mesenchymal transition (EMT) in BC and promotes metastasis (1). Bolsetti et al. reported on the unique role of the MToR pathway modulation by using drug repurposing. Metformin, a well-known hypoglycemic drug, has been shown to affect BC cell metabolism and cancer hallmarks, such as EMT. 

BC has been explored in studies ranging from metabolomics to analyze biomarker discovery (Yang et al.) and metastases (Yang et al.) to the impact of the PNS on quality of life in patients with BC. (Yang et al.) Metabolomic studies have revealed a new dimension in understanding the metabolic impact on not just hallmarks and biomarkers of BC but also on qualitative parameters, such as psychoneurological symptoms, which have a tremendous impact on patient compliance and disease outcome. Psychoneurological symptoms are predominantly due to the psychoimmunology at the brain level, and BC patients have been shown to suffer from mental conditions more frequently than healthy controls, particularly those undergoing hormone therapy and breast reconstruction (2).

Metabolic reprogramming is a modality of tumor immune escape in cancers, and BC also exhibits a high degree of metabolic reprogramming. This Research Topic also addresses the role of metabolic reprogramming in BC progression, drug resistance, and stemness, in addition to whether epigenomic and metabolomic analyses can improve our understanding of this very important topic. A comprehensive analysis of the global literature on BC metabolic reprogramming was showcased for the first time by Huang et al. in a systematic review, which highlighted glycolytic reprogramming as a complex and effective anti-cancer target. Metastatic progression was reported to be promoted by the accumulation of glycogen, which is due to the overstimulation of gluconeogenic enzymes in MCF10a (metastatic) cells, but not in MCF10-ras (non-metastatic) cells. (Hicks et al.)

Among the metabolic signals that facilitate tumor progression and immune escape, Neu5Ac treatment has been shown to occur via lipid metabolic pathways and protein sialylation, which promote tumor progression. (Yang et al.) Sialylation masks tumor neoantigens and thus facilitates immune escape and progression. Although explored in BC, protein sialylation may also play a role in other malignancies.

Another significant metabolic alteration that is key to carcinogenesis is polyamine metabolism (PM), which is closely associated with the Tumor microenvironment (TME) and anti-tumor immunity. Wang et al. demonstrated that four unique polyamine metabolism-related genes (PMRG) are associated with BC prognosis and immunotherapy. This is a relatively understudied area, and Wang et al. comprehensively elucidated the role of PM in the BC TME and immunotherapy response.

BC diagnosis and prognosis have long been analyzed using multiple biomarkers, ranging from histopathological to liquid biopsies. The effective exploration of metabolic and epigenetic biomarkers of BC is key to the future of BC management. Among the various novel biomarkers for BC diagnosis and prognosis, Coronins are a family of highly conserved molecules that play a significant role in immune cell infiltration in the TME.

In their *in silico* analysis, Elihamu et al. demonstrated that CORO1A has good diagnostic significance due to its overexpression, with high expression associated with promoter hypomethylation. Thus, epigenetic modulation of key proteins plays a crucial role in immune infiltration of the TME and cancer progression. Similarly, the discovery of SNPs in the CCL2 and CXCL12 genes further supports the growing significance of integrated genomics and immune metabolomics (3).

Metabolomic and epigenomic alterations in the TME have also been reported to be affected by the gut microbiome across various malignancies, including colorectal, oral, hepatic, and prostate cancers. A mini-review by Oh et al. provided a quick overview of the impact of the gut microbiome across global BC studies and suggested that the use of the gut microbiome as a potential biomarker for BC is still in the preliminary phase, since the majority of the data is cross-sectional. Furthermore, future studies should focus on region-specific interventions, controlled clinical trials on pre- and probiotics, and the harmonization of methods for sampling, sequencing, and analysis. Currently, evidence for the strong impact of the gut microbiome on BC metabolomics and epigenomics is lacking.

The discovery of novel salivary biomarkers was reported by Jiang et al. Although with a small sample size, the authors validated 2-aminonicotinic acid and theobromine as promising non-invasive biomarkers for BC detection.

Another foundation of this Research Topic is technological innovation. Multimodal MRI was used to predict HER2 expression through the use of deep learning, an important development in non-invasive precision oncology. This method can be an example of how AI can fill the gap between clinical practice and molecular biology by combining imaging, computational modeling, and clinical decision tools. However, concerns about model interpretability, reproducibility, and clinical integration are still significant (4).

The integration of metabolomics, epigenomics, genomics, and computational capabilities is enabling a more comprehensive understanding of BC biology. However, data integration between platforms, standardization of methodologies, and translation of findings into clinical practice still represent a significant challenge. 

We emphasize the importance of interdisciplinary collaboration, advanced validation frameworks, and patient-centered approaches. We believe that an understanding of epigenomics and metabolomics should be used to transform the future of diagnostic and therapeutic approaches in BC. Although the bench-to-bedside experience is complex, the studies compiled here provide a solid basis for realizing the promise of precision oncology in BC, with an exciting future.
