# Correction: Editorial: Epigenomics and metabolomics: pioneering new frontiers in breast cancer research and treatment

**DOI:** 10.3389/fonc.2026.1885867

**Published:** 2026-06-10

**Authors:** Purvi Purohit, Parmod Kumar, Anupama Modi

**Affiliations:** 1 Department of Biochemistry, All India Institute of Medical Sciences Jodhpur, Jodhpur, India; 2 Department of Medical Oncology Hematology, All India Institute of Medical Sciences Jodhpur, Jodhpur, India; 3 GTU-School of Applied Sciences and Technology, Ahmedabad, India

**Keywords:** breast cancer, epigenomics, metabolomics, precision medicine, TME (tumor microenvironment)

Affiliation “Department of Medical Oncology Hematology, All India Institute of Medical Sciences Jodhpur, Jodhpur, India” was omitted for author Parmod Kumar. This affiliation has now been added for author Parmod Kumar.

Author Parmod Kumar was erroneously assigned to affiliation “Department of Biochemistry, All India Institute of Medical Sciences Jodhpur, Jodhpur, India”. This affiliation has now been removed for author Parmod Kumar.

Author Anupama Modi was erroneously assigned to affiliation “Department of Medical Oncology, Gujarat Technological University, Ahemdabad, India”. This affiliation has now been removed for author Anupama Modi.

Affiliation “GTU-School of Applied Science and Technology, Ahemdabad, India” was omitted from the paper. This affiliation has now been added for author Anupama Modi.

The original version of this article has been updated.

